# Impact of Dietary Fatty Acid Composition on the Intestinal Microbiota and Fecal Metabolism of Rats Fed a High-Fructose/High-Fat Diet

**DOI:** 10.3390/nu16213774

**Published:** 2024-11-03

**Authors:** Zhihao Zhao, Lihuang Zhong, Pengfei Zhou, Yuanyuan Deng, Guang Liu, Ping Li, Jiarui Zeng, Yan Zhang, Xiaojun Tang, Mingwei Zhang

**Affiliations:** Sericultural & Agri-Food Research Institute, Guangdong Academy of Agricultural Sciences/Key Laboratory of Functional Foods, Ministry of Agriculture and Rural Affairs/Guangdong Key Laboratory of Agricultural Products Processing, Guangzhou 510610, China; zhaozhihao1991@163.com (Z.Z.);

**Keywords:** fatty acid, intestinal microbiota, metabolism, proteolytic fermentation, insulin resistance

## Abstract

**Background/Objectives**: An inappropriate intake of dietary fats can disrupt the homeostasis of intestinal microbiota, affect the host’s metabolic status, and increase the risk of chronic diseases. The impact of dietary fat types on the composition and metabolic functionality of the intestinal microbiota has become a research focus over recent years. The objective of this study was to explore the effects of regular peanut oil (PO) and high-oleic-acid peanut oil (HOPO) on the composition and metabolic function of the intestinal microbiota. **Methods**: A dietary intervention test was conducted on SD rats fed a high-fat/high-fructose (HFF) diet. The composition and metabolic functionality of the intestinal microbiota of the experimental rats were investigated by 16S rRNA gene sequencing and fecal metabolomics. **Results**: Compared with saturated fat, PO and HOPO enhanced the diversity of intestinal microbiota in HFF diet-fed rats. Compared with PO, HOPO significantly increased the relative abundance of *Lachnospiraceae_NK4A136_group* and *Harryflintia* (*p* < 0.05), which are able to generate butyrate and acetate. Compared with saturated fat, 318 and 271 fecal biomarkers were identified in PO and HOPO groups, respectively. In contrast, 68 fecal biomarkers were identified between the PO and HOPO groups. The inhibition of harmful proteolytic fermentation in the colon may represent the main regulatory mechanism. With regard to metabolic status, HOPO provided better control of body weight and insulin sensitivity than PO. **Conclusions**: Compared with saturated fat, peanut oils better regulated the composition and metabolic function of the intestinal microbiota. In addition, HOPO exhibited better regulatory effects than PO.

## 1. Introduction

The human intestinal microbiota consists of trillions of microorganisms that inhabit the gastrointestinal tract. These microorganisms express a 150-fold higher number of genes than human cells [1]. The intestinal microbiota varies in abundance and composition in different locations of the gastrointestinal tract and performs different functions [2]. Overall, the intestinal microbiota performs a crucial role in sustaining normal physiology and nutritional status through a highly mutualistic symbiotic relationship, including its ability to produce vitamins and convert indigestible fiber into a form usable for human metabolism [3,4]. Furthermore, the intestinal microbiota provides protection against external pathogens and contributes to preserving the stability of the intestinal epithelial barrier [5,6]. An increasing body of research highlights the strong connection between the intestinal microbiota and the health status of a given host, particularly with regard to nutritional and metabolic disorders, including obesity, metabolic syndrome, and diabetes [7]. Consequently, managing disruptions in the intestinal microbiota has become a promising approach for preventing and treating related diseases [8].

The human intestinal microbiota exhibits significant variability among individuals. Genetics, age, diet, medications, and various environmental factors are all known to influence the specific composition of the intestinal microbiota [9,10,11]. Regarding dietary patterns, a substantial body of evidence now connects dietary fats, the composition of the intestinal microbiota, and metabolic disorders. Obesity-related dysbiosis is known to be directly associated with a high-fat diet and is characterized by a lower overall microbiota count, changes in the abundance of bacterial species, and an overall increase in gut permeability [12,13]. Moreover, specific types of fatty acids can influence the composition of the microbiota regardless of fat consumption levels; this includes a range of factors, including the degree of saturation, carbon chain length, and double bond position [14]. The potential mechanisms may involve influencing bacterial fatty acid synthesis and interacting with bile salts [15,16].

Saturated fat has been used as a fat source in previous studies of high-fat diets [12,17,18]; however, the main aim of these earlier studies was not to investigate the specific effects of this type of fat [14]. A common observation is that a diet rich in saturated fatty acids (SFAs) tends to reduce the diversity [19] and richness [20] of the intestinal microbiota, reduce the abundance of *Bacteroidetes*, and increase the abundance of *Firmicutes*, thus mirroring the response observed in mice consuming a high-fat diet [20,21]. Diets rich in polyunsaturated fatty acids (PUFAs), such as fish oil or safflower oil, have demonstrated effects that contrast with those of SFA-rich diets, resulting in higher diversity [22] and a lower *Firmicutes* to *Bacteroidetes* ratio [23] in the intestinal microbiota. Previous studies have demonstrated that n-3 and n-6 PUFAs exert distinct effects on the intestinal microbiota. Unlike n-3 PUFAs, n-6 PUFAs have been associated with a reduction in anti-inflammatory *Bifidobacterium* [24] and an increase in potentially pro-inflammatory *Clostridium* [25]. Diets rich in monounsaturated fatty acids (MUFAs) have been associated with a higher abundance of *Firmicutes*, *Proteobacteria* [24], and *Bifidobacterium* [26]. A key feature of the Mediterranean diet is its high content of MUFAs in dietary fat, primarily from olive oil. A previous randomized and controlled trial revealed that the ingestion of a Mediterranean diet led to a reduction in *Prevotella* and an increase in both butyrate-producing bacteria and bacteria from the *Bifidobacteriaceae* family [27]. However, the specific impacts of various fatty acids, such as oleic acid, the predominant form of MUFA found in oil crops, on the composition and functionality of the intestinal microbiota is very poorly understood [28].

Oil seeds with high levels of oleic acid exhibit superior oxidation stability when compared to those with a high PUFA content. Furthermore, research on the Mediterranean diet has increasingly demonstrated the health benefits of oleic-acid-rich oils, including preventing obesity, maintaining glucose and lipid metabolic homeostasis, and providing antioxidant and anti-inflammatory activities [29,30,31]. In nature, only a limited number of main oil crops, predominantly olive and camellia, contain high levels of oleic acid. Consequently, increasing oleic acid content has become a major focus when breeding new varieties of oil crops. Inhibiting the conversion of oleic acid to linoleic acid by mutating fatty acid desaturase 2 (FAD2) is the primary method used to breed oil crops with high levels of oleic acid [32]. Thus far, the breeding and development of new varieties of oil crops with high levels of oleic acid has been successful for soybeans, peanuts, rapeseeds, and sunflowers. However, very few studies have investigated how various oil crops with regular or high levels of oleic acid can regulate the composition and metabolic function of the intestinal microbiota. To further investigate the effects of regular peanut oil (PO) and high-oleic-acid peanut oil (HOPO) on the structure and metabolic activity of the intestinal microbiota, we performed dietary interventions on experimental rats fed a high-fat/high-fructose (HFF) diet. The structure and function of the intestinal microbiota of the experimental rats were investigated by 16S rRNA gene sequencing and fecal metabolomics.

## 2. Materials and Methods

### 2.1. Materials

The PO (oleic acid content: 43.23%, oleic acid/linoleic acid ratio: 1.27, based on gas chromatography method) and HOPO (oleic acid content: 76.83%, oleic acid/linoleic acid ratio: 11.48, based on gas chromatography method) were purchased from Luhua Group (Laiyang, China). Food-grade fructose was obtained from Baolingbao Biology Co., Ltd. (Dezhou, China). Pharmaceutical-grade insulin injection was purchased from Novo Nordisk (China) Pharmaceuticals Co., Ltd. (Tianjin, China).

### 2.2. Animal Groups and Treatments

Forty male Sprague Dawley (SD) rats (weighing 180 ± 20 g, 6–7 weeks of age) were obtained from Guangdong Medical Laboratory Animal Center (Foshan, China). After being adaptively fed for one week, the rats were randomly assigned to four groups (n = 10), each of which followed a specific diet for 18 weeks: a normal control group (NC, normal chow diet + distilled water); a model group (M, 45% high-fat diet + 10% fructose water); a high-oleic-acid peanut oil group (HOPO, 45% high-fat diet + 10% fructose water); and a regular peanut oil group (PO, 45% high-fat diet + 10% fructose water). The main sources of fat in the high-fat diets provided to the M, HOPO, and PO groups were lard, high-oleic-acid peanut oil, and regular peanut oil, respectively. The specific formulations are detailed in Table 1. All feeds were manufactured and irradiated by Jiangsu Xietong Pharmaceutical Bio-engineering Co., Ltd. (Nanjing, China). The rats were housed in cages in a well-ventilated room under controlled conditions: a 12 h light/dark cycle, a stable temperature of 23 ± 2 °C, and a relative humidity of approximately 55 ± 5%. Weekly measurements were taken to record individual body weights, as well as their food and water intake. Fresh fecal samples were collected two days prior to sacrifice, and then transferred to a freezer at −80 °C for storage. Fasting blood-glucose tests and the insulin tolerance test (ITT) were performed one day prior to sacrifice. At the end of the dietary intervention, rats were euthanized after a 10 h fast. Blood samples were collected and then centrifuged at 4 °C at 2000× *g* for 15 min to prepare serum samples. All procedures followed the standard operating procedures set by the Animal Ethical and Welfare Committee of the Sericultural & Agri-Food Research Institute, Guangdong Academy of Agricultural Sciences.

### 2.3. Insulin Tolerance Test (ITT)

For the ITT, rats fasted for 12 h and were then given an intraperitoneal injection of insulin solution at a dosage of 0.75 U/kg body weight. Blood samples were taken from the tail vein to measure glucose levels at baseline (0 min) and at intervals of 15, 30, 60, 90, and 120 min post-injection. The measurements were taken using a glucometer (Johnson and Johnson Investment, Co., Ltd., Shanghai, China). Next, we calculated the area under the curve (AUC) to determine insulin tolerance.

### 2.4. Blood Biochemical Parameters

The serum levels of triglycerides (TG), total cholesterol (TC), low-density lipoprotein (LDL), high-density lipoprotein (HDL), insulin, TNF-a, and lipopolysaccharide binding protein (LBP) were analyzed using specific kits supplied by Nanjing Jiancheng Bioengineering Institute (Nanjing, China). Fasting blood glucose levels were measured as described in Section 2.3.

### 2.5. Homeostasis Model Assessment-Insulin Resistance (HOMA-IR)

The HOMA-IR was determined using the formula HOMA-IR = (FBG × FINS)/22.5, where FBG denotes the fasting blood glucose level and FINS indicates the fasting serum insulin level.

### 2.6. Intestinal Microbiota Analysis

Bacterial genomic DNA was extracted from fecal samples using the PowerSoil^®^ DNA Isolation Kit (MoBio, Jefferson City, MO, USA) and following the manufacturer’s guidelines. The full-length 16S rRNA gene was amplified utilizing the universal primers 27F (AGRGTTTGATYNTGGCTCAG) and 1492R (TASGGHTACCTTGTTASGACTT). The purified PCR products were then used to assess the abundance and diversity of intestinal microbiota, which was performed using the Pacbio sequencing platform. The raw subreads were corrected to obtain CCS (Circular Consensus Sequencing) sequences using SMRT Link (v8.0). Subsequently, lima software (v1.7.0) was employed to differentiate CCS sequences from various samples based on barcode sequences and to eliminate chimeras, thus resulting in high-quality CCS sequences. The sequences were then clustered into operational taxonomic units (OTUs) at a similarity level of 97% or higher using USEARCH (v10.0). OTUs with a relative abundance of <0.005% were subsequently filtered out. Alpha diversity metrics, such as ACE, Chao1, Shannon, Simpson, and PD_whole_tree, were computed for each sample. Beta diversity indices, including PCoA, ANOSIM, and ADONIS, were also determined to identify structural differences in the intestinal microbiota community between groups. The 16S rRNA gene sequencing was carried out by Biomarker Technologies Co., Ltd. (Beijing, China). Analyses of alpha diversity, beta diversity, and linear discriminant analysis effect size (LEfSe) were conducted using BMKCloud (www.biocloud.net accessed on 30 September 2024).

### 2.7. Fecal Metabolome Analysis

A 20 mg fecal sample was extracted using a 400 μL solution of methanol and water (7:3, *v*/*v*) that included an internal standard. The extracts were then analyzed with UPLC (Shim-pack UFLC Shimadzu CBM30A, Kyoto, Japan;) in conjunction with a tandem MS system (Applied Biosystems 4500 Q TRAP, Foster City, CA, USA). The UPLC was operated under the following conditions: A C18 column (1.8 μm, 2.1 mm × 100 mm) was used, with the column temperature set at 40 °C. The flow rate was maintained at 0.4 mL/min, and the injection volume was 2 μL. The solvent system consisted of water with 0.1% formic acid and acetonitrile with 0.1% formic acid. The gradient program started with a 95:5 *v*/*v* ratio at 0 min, shifted to 10:90 *v*/*v* at 11.0 min, remained at 10:90 *v*/*v* until 12.0 min, then returned to 95:5 *v*/*v* at 12.1 min, and continued at 95:5 *v*/*v* until 14.0 min. The ESI source was operated under the following conditions: The source temperature was set to 500 °C, with ion spray voltages of 5500 V for the positive mode and −4500 V for the negative mode. The pressures for ion source gas I, gas II, and the curtain gas were set at 55 psi, 60 psi, and 25 psi, respectively. Instrument tuning and mass calibration were performed with 10 and 100 μmol/L polypropylene glycol solutions in triple quadrupole (QQQ) and linear ion trap (LIT) modes, respectively. A qualitative analysis was conducted using the laboratory’s self-constructed targeted standard database, based on the retention time, ion pair information, and secondary spectral data. A quantitative analysis was performed using the multiple reaction monitoring (MRM) mode of the MS system.

For two-group analysis, significantly up- or down-regulated metabolites were identified by selecting metabolites with a VIP score of ≥1 and significance level of *p* < 0.05 in Student’s *t*-test. The identified metabolites were annotated using the KEGG Compound database (http://www.kegg.jp/kegg/compound/ accessed on 30 September 2024), and the annotated metabolites were then mapped to the KEGG pathway database (http://www.kegg.jp/kegg/pathway.html accessed on 30 September 2024). Pathways containing significantly up- or down-regulated metabolites were mapped and subsequently analyzed by Metabolite Sets Enrichment Analysis (MSEA). The significance of these pathways was assessed using *p*-values derived from the hypergeometric test.

### 2.8. Statistical Analysis

Data are shown as mean ± standard deviation (SD). Statistical analyses were conducted with SPSS software (version 22.0). Significance was evaluated using Duncan’s multiple range test, with a *p*-value < 0.05 indicating statistical significance. Origin software (version 2019b) was used for graphical analysis.

## 3. Results

### 3.1. Body Weight and Blood Physiological Index of Rats with HFF Diet

As shown in Table 2, HFF diets induced an increase in body weight, even though the four groups did not show significant differences. Compared to the NC group, a significant increase in TC, TG, HDL, LDL, FBG, ITT AUC, HOMA-IR, and LBP was observed in the M group (*p* < 0.05). Compared to the M group, HOPO had reduced body weight, TC, TG, LDL, FBG, FINS, ITT AUC, HOMA-IR, TNF-a, and LBP; however, only LDL, FINS, ITT AUC, HOMA-IR, and LBP exhibited significant differences (*p* < 0.05). Similarly, compared to the M group, PO had reduced body weight, TC, TG, LDL, FBG, FINS, ITT AUC, HOMA-IR, TNF-a, and LBP; however, only LDL, FINS, ITT AUC, HOMA-IR, and LBP exhibited significant differences (*p* < 0.05). Notably, compared to the NC group, the ITT AUC and HOMA-IR were significantly increased in the M group, thus indicating that HFF diets induced insulin resistance. However, the type of dietary fat exerted significant effects on insulin resistance. PO and HOPO reduced the ITT AUC and HOMA-IR. Specifically, HOPO exhibited better regulatory effects on insulin resistance than PO.

### 3.2. Diversity of Intestinal Microbiota

**Figure 1:**
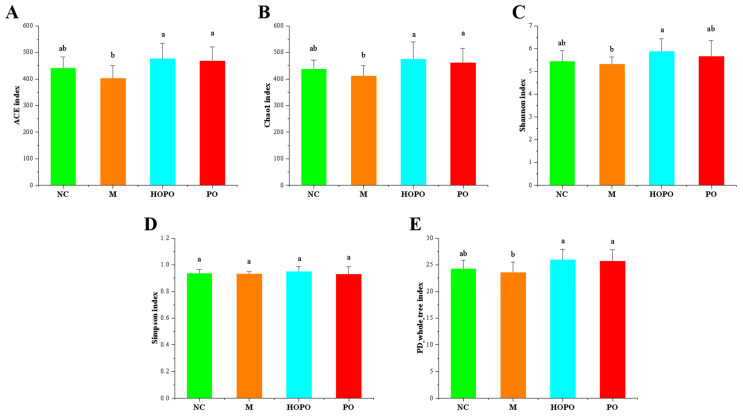
Alpha-diversity of intestinal microbiota in rats (n = 10 for each group). (**A**) ACE index. (**B**) Chao1 index. (**C**) Shannon index. (**D**) Simpson index. (**E**) PD_whole_tree index. Distinct letters indicate significant differences between groups (*p* < 0.05).

**Figure 2:**
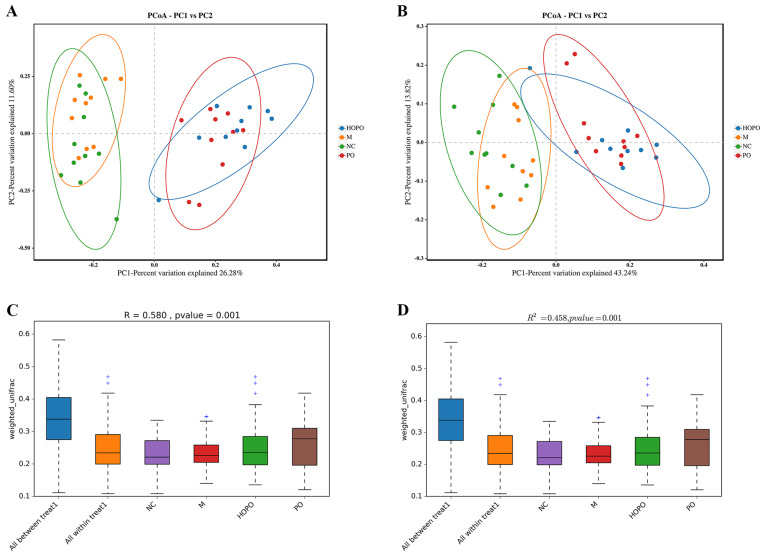
Beta-diversity of intestinal microbiota in rats (n = 10 for each group). (**A**) PCoA based on Bray–Curtis. (**B**) PCoA based on weighted Unifrac. (**C**) Anosim based on weighted Unifrac. (**D**) Adonis based on weighted Unifrac.

Alpha diversity indices, including ACE, Chao1, Shannon, Simpson, and PD_whole_tree index, were analyzed to examine the community diversity of intestinal microbiota within each sample. Compared with NC rats, rats fed an HFF diet rich in saturated fatty acids (the M group) exhibited a lower alpha diversity, which was observed in all five indices (Figure 1). In comparison to the M group, the consumption of PO and HOPO reversed this trend in alpha diversity. The consumption of PO resulted in a significant rise in the ACE, Chao1, and PD_whole_tree indices (*p* < 0.05). The consumption of HOPO led to a notable increase in the ACE, Chao1, Shannon, and PD_whole_tree indices (*p* < 0.05). Beta diversity indices, including PCoA, Anosim, and Adonis, were calculated to reflect the structural differences of intestinal microbiota between samples. As illustrated in scatter diagrams for PCoA based on Bray–Curtis and weighted-Unifrac (Figure 2A,B), there were two large overlaps between the PO and HOPO groups, as well as between the NC and M groups. Anosim analysis showed that the between-group variability of the samples was significantly greater than the within-group variability (R = 0.58, *p* < 0.01), indicating that there were significant differences in the intestinal microbiota between different groups (Figure 2C). As depicted in Figure 2D, Adonis analysis showed that the explanation degree of grouping to sample variance was 0.458, which exhibited high test reliability (*p* < 0.01). Taken together, the type of dietary fat exerted significant effects on the richness, evenness, and structural differences of the intestinal microbiota in rats fed with an HFF diet. The PO and HOPO diets enhanced the richness and evenness of the intestinal microbiota.

### 3.3. Composition of Intestinal Microbiota

**Figure 3:**
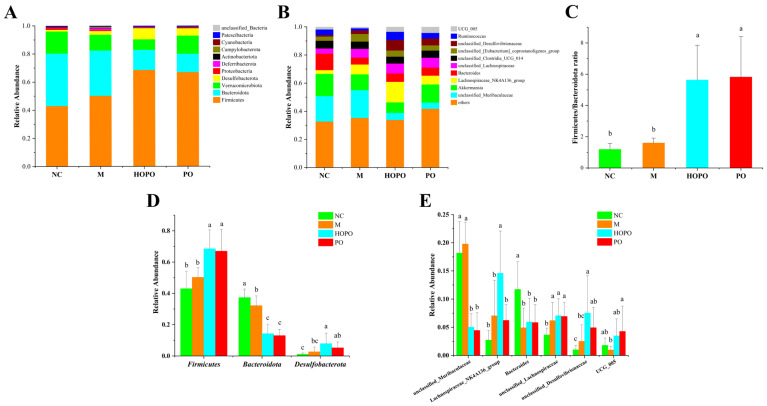
Composition of intestinal microbiota in rats (n = 10 for each group). (**A**) Relative abundance at phylum level. (**B**) Relative abundance at genus level. (**C**) *Firmicutes*/*Bacteroidetes* ratio; (**D**) Significant difference in the relative abundance between groups among all phyla. (**E**) Significant difference in the relative abundance between groups among top ten genera. Distinct letters indicate significant differences of intestinal microbiota level between groups (*p* < 0.05).

Compositions of the intestinal microbiota at the phylum level are shown in Figure 3A. *Firmicutes*, *Bacteroidota*, *Verrucomicrobiota* and *Desulfobacterota* were the dominant classifications, accounting for a total relative abundance of 96.32% to 98.24%. Relative to the NC group, *Firmicutes* showed an increased relative abundance and *Bacteroidetes* exhibited a decreased relative abundance in rats that had been fed with the three HFF diets. Of the three HFF diet groups, PO and HOPO induced a higher relative abundance of *Firmicutes* and *Desulfobacterota* than observed in the M group; this was accompanied by a reduction in the relative abundance of *Bacteroidota*. Analysis of variance (ANOVA) further revealed that these three phyla exhibited significant differences in the relative abundance between groups across all phyla (Figure 3D). Compared with the M group, the HOPO group showed significantly higher relative abundances of *Firmicutes* and *Desulfobacterota*, and a significantly lower relative abundance of *Bacteroidota* (*p* < 0.05). There was no significant difference in the relative abundance of *Desulfobacterota* between the M and PO groups. None of the phyla exhibited a significant difference in relative abundance when compared between the PO and HOPO groups. As shown in Figure 3C, the *Firmicutes*/*Bacteroidetes* ratio for the PO and HOPO groups was significantly higher than that of the NC and M groups (*p* < 0.05).

The top ten species, at the genus level, identified in the intestinal microbiota are shown in Figure 3B. The dominant genera in the NC group were *unclassified_Muribaculaceae*, *Akkermansia*, and *Bacteroides*. The HFF diets induced a reduction in the abundance of *Akkermansia* and an increase in the abundance of *Lachnospiraceae_NK4A136_group*, *unclassified_Lachnospiraceae*, and *unclassified_Desulfovibrionaceae*. ANOVA revealed that six of the top ten genera exhibited significant between-group differences (Figure 3E). Compared with the NC group, the M group exhibited a significantly lower relative abundance of *Bacteroides*, and a significantly higher relative abundance of *unclassified_Lachnospiraceae*. The PO and HOPO groups exhibited a higher abundance of *Bacteroides* than the M group, although these groups did not display significant differences. Compared with the M group, the PO group exhibited a significantly lower relative abundance of *unclassified_Muribaculaceae* and a significantly higher relative abundance of *UCG_005* (*p* < 0.05). The HOPO group exhibited a significant reduction in the abundance of *unclassified_Muribaculaceae* level (*p* < 0.05) and a significant rise in the abundance of *Lachnospiraceae_NK4A136_group, unclassified_Lachnospiraceae*, and *unclassified_Desulfovibrionaceae* (*p* < 0.05).

### 3.4. LEfSe Analysis of Intestinal Microbiota

**Figure 4:**
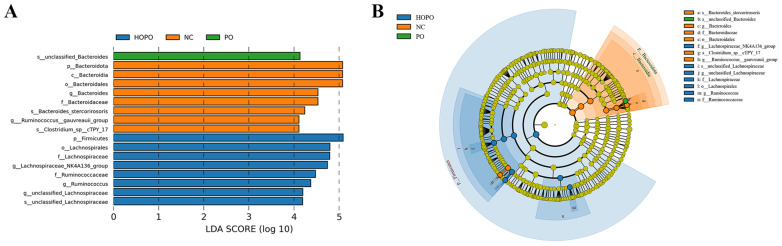
LEfSe analysis of intestinal microbiota in rats (n = 10 for each group). (**A**) LDA distribution displays species with significant abundance (LDA > 4) across different groups. (**B**) Hierarchical tree for different groups. The outward-radiating circles depict taxonomic levels ranging from phylum to species. The small circle at each specific level indicates a classification, with its diameter reflecting the species’ relative abundance.

Linear discriminant analysis effect size (LEfSe) is a method used to identify features in high-dimensional data that are both statistically significant and biologically relevant. LEfSe is capable of handling multi-level categorical data and identifying features that play a role in differentiating groups. The results of the LEfSe analysis of the intestinal microbiota across different groups are provided in Figure 4, with the LDA threshold set to 4.0. In total, seventeen bacterial species exhibited significant differences between the four groups (Figure 4A). Eight distinct species were more abundant in the NC group: *Bacteroidota*, *Bacteroidia*, *Bacteroidales*, *Bacteroides*, *Bacteroidaceae*, *Bacteroides_stercorirosoris*, *Ruminococcus__gauvreauii_group*, and *Clostridium_sp__cTPY_17*. Eight distinct species were more abundant in the HOPO group: *Firmicutes*, *Lachnospirales*, *Lachnospiraceae*, *Lachnospiraceae_NK4A136_group*, *Ruminococcaceae*, *Ruminococcus*, *unclassified_Lachnospiraceae* (genus), and *unclassified_Lachnospiraceae* (species). Analysis identified *unclassified_Bacteroides* as the only dominant bacteria in the intestinal microbiota in the PO group. Construction of a hierarchical tree revealed that the dominant bacteria in the NC group were mainly *Bacteroidetes*, while the dominant bacteria in the HOPO group were mainly *Firmicutes* (Figure 4B). However, no dominant type of bacteria was identified in the M group.

### 3.5. Analysis of Fecal Metabolome Profile

**Figure 5:**
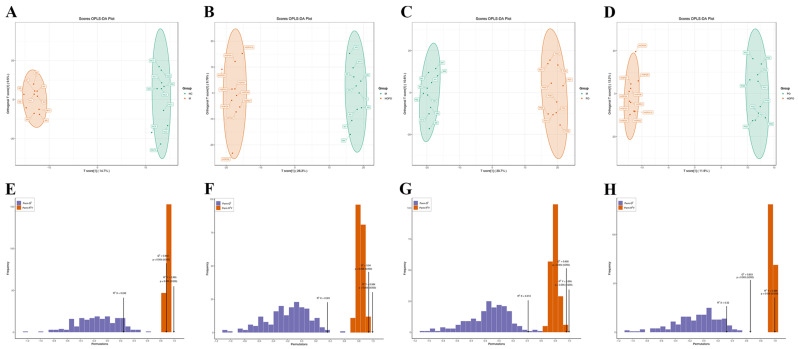
OPLS-DA scoring map and model validation of fecal metabolome profile (n = 10 for each group). (**A**) Scoring map between NC and M groups. (**B**) Scoring map between M and HOPO groups. (**C**) Scoring map between M and PO groups. (**D**) Scoring map between PO and HOPO groups. (**E**) Model validation between NC and M groups. (**F**) Model validation between M and HOPO groups. (**G**) Model validation between M and PO groups. (**H**) Model validation between PO and HOPO groups.

The effect of dietary fatty acid composition on the fecal metabolome of rats fed a high-fat diet was evaluated using UPLC-MS/MS. As demonstrated in Figure 5A–D, the metabolic profiles were completely separated into different groups based on the OPLS-DA model (NC vs. M, M vs. HOPO, M vs. PO, and PO vs. HOPO). R2Y values were all >0.99 (*p* < 0.005), indicating the excellent explanatory efficiency of the OPLS-DA model. In addition, Q2 values were >0.65 (*p* < 0.005), indicating the good predictive efficiency of the OPLS-DA model (Figure 5E–H). Collectively, these results demonstrate that the OPLS-DA models could identify differential metabolites between various groups in this study. These findings suggest that diets with varying fatty acid compositions result in unique fecal metabolic profiles. The content of oleic acid in PO led to significant changes in the fecal metabolome of rats fed with an HFF diet.

### 3.6. Metabolic Fecal Biomarkers and Pathway Enrichment

**Figure 6:**
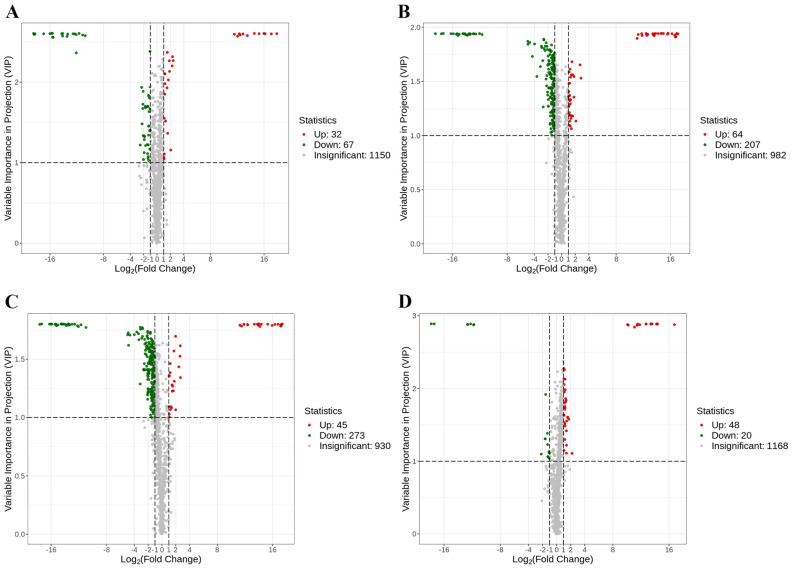
Volcano plots of fecal metabolome (n = 10 for each group). (**A**) Volcano plots between NC and M groups. (**B**) Volcano plots between M and PO groups. (**C**) Volcano plots between M and PO groups. (**D**) Volcano plots between PO and HOPO groups.

Volcano plots were generated to illustrate the altered metabolites across different groups by employing a combined screening method using VIP values and fold changes (VIP > 1, fold change > 2 or fold change < 0.5). As shown in Figure 6A–D, there were 99, 271, 318, and 68 fecal biomarkers identified between the NC and M group, M and HOPO group, M and PO group, and PO and HOPO group, respectively.

**Figure 7:**
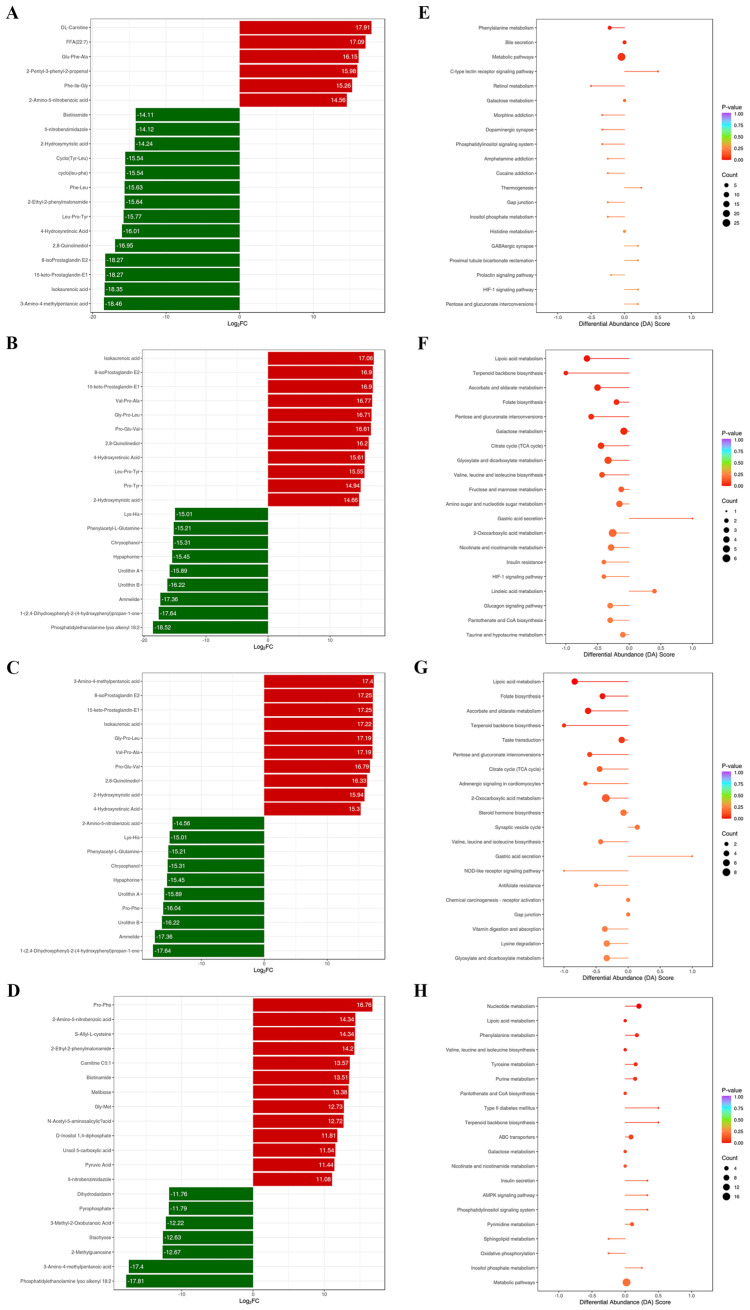
Metabolic fecal biomarkers and enrichment of pathways (n = 10 for each group). (**A**–**D**) The top 20 metabolites with the largest fold changes between NC and M groups, M and HOPO groups, M and PO groups, and PO and HOPO groups, respectively. (**E**–**H**) Differential Abundance Score based on KEGG between NC and M groups, M and HOPO groups, M and PO groups, PO and HOPO groups, respectively.

Compared with the NC group, the relative levels of DL-Carnitine, FFA (22:7), Glu-Phe-Ala, 2-Pentyl-3-phenyl-2-propenal, Phe-Ile-Gly, and 2-Amino-5-nitrobenzoic acid increased in the M group among the top 20 metabolites by fold change, while the relative levels of Biotinamide, 5-nitrobenzimidazole, 2-Hydroxymyristic acid, Cyclo(Tyr-Leu), Cyclo(leu-phe), Phe-Leu, 2-Ethyl-2-phenylmalonamide, Leu-Pro-Tyr, 4-Hydroxyretinoic Acid, 2,8-Quinolinediol, 8-isoProstaglandin E2, 15-keto-Prostaglandin-E1, Isokaurenoic acid, and 3-Amino-4-methylpentanoic acid decreased (Figure 7A). Biomarkers between the NC group and M group were mainly concentrated in pathways associated with amino acid metabolism, carbohydrate metabolism, vitamin metabolism, and synapse systems (Figure 7E).

Compared with the M group, the relative levels of Isokaurenoic acid, 8-isoProstaglandin E2, 15-keto-Prostaglandin-E1, Val-Pro-Ala, Gly-Pro-Leu, Pro-Glu-Val, 2,8-Quinolinediol, 4-Hydroxyretinoic Acid, Leu-Pro-Tyr, Pro-Tyr, and 2-Hydroxymyristic acid in the HOPO group were increased among the top 20 metabolites by fold change, while the relative levels of Lys-His, Phenylacetyl-L-Glutamine, Chrysophanol, Hypaphorine, Urolithin A, Urolithin B, Ammelide, 1-(2,4-Dihydroxypheny)-2-(4-hydroxyphenyl)propan-1-one, and Phosphatidylethanolamine lyso alkenyl 18:2 decreased (Figure 7B). The biomarkers between the M and HOPO groups were mainly concentrated in pathways associated with energy, amino acid, carbohydrate, and vitamin metabolism, as well as the insulin signal pathway. Most notably, the pathways associated with insulin resistance and the glucagon signaling pathway were downregulated in the HOPO group (Figure 7F).

Compared with the M group, the relative levels of 3-Amino-4-methylpentanoic acid, 8-isoProstaglandin E2, 15-keto-Prostaglandin-E1, Isokaurenoic acid, Gly-Pro-Leu, Val-Pro-Ala, Pro-Glu-Val, 2,8-Quinolinediol, 2-Hydroxymyristic acid, and 4-Hydroxyretinoic Acid in the PO group were increased among the top 20 metabolites by fold change, while the relative levels of Amino-5-nitrobenzoic acid, Lys-His, Phenylacetyl-L-Glutamine, Chrysophanol, Hypaphorine, Urolithin A, Pro-Phe, Urolithin B, Ammelide, and 1-(2,4-Dihydroxyphenyl)-2-(4-hydroxyphenyl)propan-1-one decreased (Figure 7C). Biomarkers between the M and PO groups were mainly concentrated in pathways associated with energy metabolism, amino acid metabolism, carbohydrate metabolism, and vitamin metabolism (Figure 7G).

Next, the differential metabolites and enriched pathways between the HOPO and PO groups were comparatively analyzed to directly investigate the impact of dietary fatty acid composition on the fecal metabolome. Compared with the PO group, the relative levels of Pro-Phe, 2-Amino-5-nitrobenzoic acid, S-Allyl-L-cysteine, 2-Ethyl-2-phenylmalonamide, Carnitine C5:1, Biotinamide, Melibiose, Gly-Met, N-Acetyl-5-aminosalicylic acid, D-Inositol 1,4-diphosphate, Uracil 5-carboxylic acid, Pyruvic Acid, and 5-nitrobenzimidazole in HOPO group were increased among the top 20 metabolites by fold change, while the relative levels of Dihydrodaidzein, Pyrophosphate, 3-Methyl-2-Oxobutanoic acid, Stachyose, 2-Methylguanosine, 3-Amino-4-methylpentanoic acid, and Phosphatidylethanolamine lyso alkenyl 18:2 decreased (Figure 7D). Biomarkers between the PO and HOPO groups were mainly concentrated in pathways associated with energy metabolism, lipid metabolism, and amino acid metabolism. Most notably, the pathways associated with insulin secretion showed alteration (Figure 7H).

## 4. Discussion

Numerous studies have investigated the effects of dietary fat quantity and quality on the composition of the intestinal microbiota and metabolic health. As a characteristic of unhealthy Western-style diets, high fat consumption has been linked to a reduced richness and diversity in the intestinal microbiota in both animals and humans [20,33,34]. The influence of fatty acid types on the composition of the intestinal microbiota and metabolic health is less comprehensively understood than the effects of high fat consumption. In such studies, dietary fat has typically been sourced from oil crops, animals, and microalgae, which exhibit distinct differences in their fatty acid composition. In addition to variations in fatty acid composition, the concomitant phytochemicals present in different fat sources are also known to differ significantly. The primary phytochemical in extra virgin olive oil, which is rich in MUFAs, is squalene, whereas in flaxseed oil, which is rich in PUFAs, the primary phytochemical is phytosterol [35]. However, these minor phytochemical components in dietary fat can cause distinct alterations in the structure and function of the intestinal microbiota [36,37,38]. To some extent, this has impeded the progress of related research, leading to less consistent findings regarding the specific effects of diets rich in MUFAs [39]. Therefore, in the present study, we investigated the modulatory effects of MUFA-rich fats on the intestinal microbiota using high-oleic peanut oil. Additionally, the study included a control group with regular peanut oil that had a moderate oleic acid content, thereby reducing the interference from natural phytochemical components present in the oils.

Previous research indicated that, in contrast to the lower diversity of the intestinal microbiota induced by SFAs, the effects of MUFA-rich diets are less consistent. MUFAs appear to have no impact on the richness and diversity of the intestinal microbiota of mice [21]. Furthermore, diets that are high in MUFAs are known to exert adverse impacts on the richness and diversity of the intestinal microbiota in populations at high risk for metabolic syndrome [40]. Additionally, in a cross-sectional study, consuming a high amount of MUFAs was found to be negatively correlated with the diversity and richness of the intestinal microbiota [41]. In the present study, both PO and HOPO were found to significantly enhance the richness and diversity of the intestinal microbiota compared to SFA, while showing no significant difference compared to the NC group. There was no significant difference in the richness and diversity of intestinal microbiota when comparing the PO and HOPO groups. This result may support the conclusion that, compared to a regular diet, a diet rich in MUFAs might not have a significant impact on intestinal microbiota diversity. An increase in the *Firmicutes*/*Bacteroidetes* ratio is often cited as an indicator associated with obesity. However, the literature presents numerous contradictory results, making it difficult to establish a definitive link between the *Firmicutes*/*Bacteroidetes* ratio and specific health outcomes [42]. The *Firmicutes*/*Bacteroidetes* ratio was found to be significantly higher in the PO and HOPO groups in the present study. Nonetheless, differences in the composition of the intestinal microbiota were observed between these two groups. Although the relative abundance of *Firmicutes* and the *Firmicutes*/*Bacteroidetes* ratio did not differ significantly between the two peanut oil groups, compared with PO, HOPO significantly increased the relative abundance of *Lachnospiraceae_NK4A136_group* and *Harryflintia*, which are members of the family *Lachnospiraceae* and phylum *Firmicutes,* at the genus level. The *Lachnospiraceae_NK4A136_group* is able to generate a vital type of short-chain fatty acid known as butyrate, which serves as a vital energy source for colon epithelial cells, while also playing a role in inhibiting the release of inflammatory cytokines and enhancing epithelial barrier integrity by upregulating protein expression in tight junctions [43]. Therefore, this is a beneficial microorganism that plays an important role in maintaining intestinal homeostasis and has been found to alleviate inflammatory bowel disease [44]. *Harryflintia*, a newly identified genus within the *Lachnospiraceae* family, is postulated to contribute to carbohydrate fermentation and acetate production [45].

Metabolites of the intestinal microbiota, including amino acids, short-chain fatty acids, vitamins, bile acids, and their derivatives, play significant roles in modulating physiological and immune functions in their host, and their balance can exert direct impacts on health status [46]. The microbial metabolism of carbohydrates, proteins, and primary bile acids in the gut can generate bioactive metabolites that are not directly digested or absorbed from dietary intake [47]. In this study, a large number of fecal biomarkers were identified between the M and HOPO groups, and between the M and PO groups (271 and 318 metabolites, respectively). In contrast, a smaller number of fecal biomarkers, totaling 68 metabolites, were identified between the PO and HOPO groups. Compared to an SFA-rich diet, the intake of PO and HOPO led to a greater similarity in both the intestinal microbiota composition and fecal metabolite profiles. Compared to the M group, the PO and HOPO groups exhibited an increase in the levels of several peptides and a reduction in the levels of phenylacetyl-L-glutamine; these changes were both identified among the top 20 metabolites with the largest fold changes. Phenylacetyl-L-glutamine, a metabolite produced by the intestinal microbiota through the fermentation of residual peptides and proteins in the distal colon [48], has been associated with multiple disease risks, including cardiovascular diseases, type 2 diabetes, acute myocardial infarction, and Parkinson’s disease [49,50,51]. The intestinal microbiota in the distal colon primarily utilizes residual peptides and proteins for energy through proteolytic fermentation, as fermentable carbohydrates are already consumed in the proximal colon. Notably, proteolytic fermentation produces a wide variety of metabolites in the distal colon, which are often regarded as harmful to gut integrity and metabolic health [52]. These contrasting changes in the levels of peptides and phenylacetyl-L-glutamine suggest that both PO and HOPO may inhibit proteolytic fermentation in the distal colon by modulating the composition of the intestinal microbiota. This form of regulation potentially promotes metabolic health and intestinal homeostasis by reducing the production of harmful metabolites. Furthermore, in both the PO and HOPO groups, inhibition of the BCAA (valine, leucine, and isoleucine) biosynthesis pathway was observed. This pathway is known to be positively associated with protein fermentation in the colon [53,54]. It is reasonable to hypothesize that the inhibition of proteolytic fermentation in the distal colon may be the main metabolic regulatory mechanism involved.

This study has several limitations. While the mechanisms by which diet influences host metabolic health are diverse, our research focused exclusively on the structure and function of the gut microbiota. As a result, potential molecular mechanisms such as the regulation of glucose and lipid metabolic pathways were not explored. Future research should aim to investigate these mechanisms more thoroughly and conduct dietary intervention experiments specifically targeting patients with nutritional metabolic diseases.

## 5. Conclusions

In summary, this study confirms that the type of dietary fat can affect the composition and metabolic functionality of the intestinal microbiota in rats fed with an HFF diet. Substituting lard with peanut oil (PO and HOPO) alleviated a number of metabolic disorders in rats, including obesity, dyslipidemia, and insulin resistance. The primary mechanism appears to be the suppression of proteolytic fermentation in the distal colon. Specifically, peanut oil derived from high-oleic-acid varieties offers better metabolic regulation of the composition and functionality of the intestinal microbiota than regular peanut varieties.

## Figures and Tables

**Table 1:** The formulations of the diets.

	NC	M	HOPO	PO
Caloric ratio/% kcal				
Fat	10	45	45	45
Protein	20	20	20	20
Carbohydrate	70	35	35	35
Fat source/% weight				
HOPO	—	—	11.8	—
PO	—	—	—	11.8
Soybean oil	2.4	2.9	2.9	2.9
Lard	1.9	20.7	8.9	8.9

**Table 2:** Physiological and serum biochemical parameters of rats in each group.

	NC	M	HOPO	PO
Body weight (g)	667.70 ± 48.26	701.90 ± 67.12	669.30 ± 39.80	681.00 ± 83.14
TC (mmol/L)	2.03 ± 0.36 b	2.73 ± 0.51 a	2.42 ± 0.44 ab	2.50 ± 0.47 a
TG (mmol/L)	0.88 ± 0.35 b	1.24 ± 0.44 a	1.15 ± 0.22 ab	1.05 ± 0.33 ab
HDL (mmol/L)	0.77 ± 0.17 b	1.04 ± 0.18 a	1.09 ± 0.21 a	1.14 ± 0.23 a
LDL (mmol/L)	0.73 ± 0.12 c	1.12 ± 0.27 a	0.92 ± 0.16 c	1.03 ± 0.19 ab
FBG (mmol/L)	5.87 ± 0.73 b	6.88 ± 0.61 a	6.41 ± 0.98 ab	6.49 ± 0.73 ab
FINS (mIU/L)	7.53 ± 1.14 ab	8.56 ± 1.44 a	5.90 ± 1.58 c	7.08 ± 1.24 bc
ITT AUC	482.48 ± 59.28 b	554.78 ± 67.81 a	488.18 ± 51.91 b	513.45 ± 73.00 ab
HOMA-IR	1.96 ± 0.34 b	2.62 ± 0.54 a	2.05 ± 0.45 b	1.66 ± 0.40 b
TNF-a (ng/L)	85.99 ± 12.01	97.22 ± 11.92	86.53 ± 8.58	91.58 ± 13.04
LBP (ng/L)	64.65 ± 1.69 c	69.04 ± 1.86 a	64.93 ± 1.01 c	67.02 ± 1.83 b

Data are presented as means ± SD (n = 10 for each group). Distinct letters on the same line indicate significant differences between groups (*p* < 0.05, based on Duncan’s multiple range test).

## Data Availability

The original contributions presented in the study are included in the article, further inquiries can be directed to the corresponding author.
